# Could Emotional Intelligence Ability Predict Salary? A Cross-Sectional Study in a Multioccupational Sample

**DOI:** 10.3390/ijerph18031322

**Published:** 2021-02-01

**Authors:** Martin Sanchez-Gomez, Edgar Breso, Gabriele Giorgi

**Affiliations:** 1 Department of Evolutionary, Educational, Social Psychology and Methodology, Universitat Jaume I, 12071 Castellón de la Plana, Spain; breso@uji.es; 2 Department of Human Sciences, European University of Rome, 00163 Rome, Italy; gabriele.giorgi@unier.it

**Keywords:** emotional intelligence, salary, job success, performance, salary, workplace

## Abstract

The study of emotional intelligence (EI) in work environments is a trending topic. However, few studies have examined the relationship between EI and salary. Therefore, the presented research aims to analyze the influence of EI on salary using a multioccupational sample. The participants were 785 subjects aged between 18 and 58 years (M = 39.41; SD = 10.95). EI ability was measured using the Mobile Emotional Intelligence Test (MEIT), while the salary was collected together with other sociodemographic variables in a questionnaire created ad hoc. After controlling for the age, gender, social class, educational level, and work experience variables, the results of correlation and regression analysis showed that participants with higher EI and emotional-repair capacity generally have higher salary. These findings provide preliminary evidence that EI is a relevant variable in achieving career success. The ability to channel and manage emotions could help employees develop stronger interpersonal relationships, leading to higher positions and greater financial compensation.

## 1. Introduction

### 1.1. Emotional Intelligence at the Workplace

Since the term emotional intelligence (EI) was introduced three decades ago [1], many studies have been carried out in this field. Mayer and Salovey [2] defined EI as a mental ability, suggesting that emotionally intelligent people perceive emotions more accurately. This enables them to use emotions to facilitate thinking, to understand them and their meanings, and to manage their own emotions as well as someone else’s in a more optimal way. In a nutshell, EI can be defined as the meta-ability to perceive and understand emotions, as well as to manage them appropriately and adaptively [3].

Research has shown that there are significant differences between individuals in terms of their emotional abilities—especially when using affective information and integrating it into their behavior—which has important consequences at different levels [4]. For example, at an individual level it has been related to well-being [5] and mental health [6]; at a social level, it has been related to group acceptance [7] and psychosocial adjustment [8]; and at a professional level, to leadership [9], active job searches [10], satisfaction [11], intention to quit [12], psychological distress [13], and professional success [14,15], among others.

Taking into account the relevance of emotional skills in the workplace, creating interpersonal connections at work has become increasingly important in today’s global environment, where jobs are dynamic and require easy access to information and strong social and managerial support [14,16]. Various studies suggest that the regulatory processes associated with EI can benefit people’s social relationships since the correct use of emotion and being aware of one’s own emotions can lead to better stress regulation and higher performance [17,18]. McFarland et al. [19] found out that employees with high EI had a better job performance, a longer-term perspective, and lower levels of burnout under conditions of high-role stress. For their part, Armstrong et al. [20] consider EI an important predictor of professional success, partly due to its role in building interpersonal relationships and developing effective coping strategies. Nguyen et al. observed that both self-emotion appraisal and others’ emotion appraisal precede emotion regulation, leading to a positive effect on job performance [17]. In this sense, Rode et al. [21] argue that EI is essential for acquiring the social support necessary to achieve a successful career.

### 1.2. Emotional Intelligence and Salary

Recent research studies have focused on the relationship between EI and job success, taking salary as an objective measure. The study carried out by Momm et al. [14] indicates, on the one hand, that the ability to recognize emotions is linked to annual salary and, on the other, that this relationship is mediated by two variables: political ability and interpersonal facilitation. In the same way, Côté et al. [22] found that people who better regulate their emotional reaction have higher salary and socioeconomic status. Rode et al. [21], after controlling gender and age variables, also point out that interpersonal and leadership skills help to achieve greater financial compensation. In addition, they found that the effects of EI on salary are more extensive at the highest organizational levels. However, this significant relationship between salary and EI has also been observed at the beginning of the working career [23]. Haro et al. [24] suggested that salary was indirectly predicted by personality traits, such as neuroticism (both positively and negatively), extraversion (positively), and openness (positively) via the EI dimensions, following the causal chain: perception, understanding, and emotional regulation. Despite all this research, scientific literature has not reached a consensus on this particular matter, since several authors have questioned the relationship between EI and salary. Antonakis et al. [25], as well as Joseph and Newman [26] observed that EI was not able to explain the variability in job performance or job success. In the same vein, Rode et al. [27] did not find a significant relationship between EI and salary when studying this relationship in subjects with two years of work experience. Therefore, it seems that the findings are still confusing, which highlights the necessity to further research this topic.

### 1.3. Covariables

Following similar previous studies [14,21,28], personal, economic and demographic factors that could represent alternative explanations for the observed effects were included.

On the one hand, gender and age has shown a strong relationship with EI and salary. Regarding gender, the results suggest that it affects the ability EI score, with the EI being greater in women than men [29]. However, women earn less money than men on average, although this could be explained through other variables as academic rank [30]. According to Rode [21], gender has shown a suppression effect on the relationship between EI and salary. Regarding age, EI followed an inverted-U curve: younger and older adults scored lower on ability EI than middle-aged adults [29]. In addition to this, previous research [21,23,28] found proof of the influence of age and years of experience on salary, that is older workers with more experience are those with a higher salary.

On the other hand, several socioeconomic variables have been linked to EI and salary. Among them, we highlight educational level and social class. These variables reflect job autonomy and wider social capital, which provide more economic opportunities [31]. For example, the positive influence of the educational level on salary can be observed in reports made by the Spanish National Institute of Statistics [32]. Moreover, we cannot dismiss the possibility that participants who belong to a higher social class have higher emotional intelligence and, in turn, earn more money. Hence, it is important to assess all these variables to find out if EI predicts salary above other variables that could theoretically predict it. Without controlling the effect of these variables, it is difficult to know the unique contribution that EI makes to the variance of salary.

### 1.4. The Present Study

Emotional intelligence should help employees in the development of their professional career, leading to a higher financial compensation. Thus, the present study aims to analyze whether EI predicts the salary. To test this relationship, it was examined whether an ability-based measure of emotional intelligence in a multioccupational sample predicts the salaries of the samples, when controlled by age, gender, social class, educational level, and professional experience. We hypothesize that there is a positive relationship between EI and salary. Furthermore, we expected to find a significant relationship between salary and age, gender, social class, educational level, and professional experience, based on previous findings.

## 2. Materials and Methods

### 2.1. Sample

The present research follows a cross-sectional design. The participants in this study were 785 subjects (39.1% men and 60.9% women) between 18 and 58 years of age (M = 39.41, SD = 10.95).

The educational level of the sample was divided into higher studies (i.e., university degree, master, and PhD) (58.2%), intermediate studies (i.e., upper secondary education and vocational training) (29.7%), and basic studies (i.e., school graduate and secondary education) (12.1%). On the other hand, participants declared themselves as belonging to different social classes such as lower (14.3%), middle (80.2%), and upper (5.5%). The most representative sectors were administration (24.6%), education (23.5%) and healthcare (18.3%), while almost half of the sample (47.6%) were employed in the private sector. For detailed information about individual characteristics of the participants, see Table 1.

### 2.2. Instruments

#### 2.2.1. Emotional Intelligence

To assess EI, the MEIT [33] was used. This questionnaire is based on the ability model of Salovey and Mayer [2] and follows the model of skill tests with correct and incorrect answers, similar to the intelligence quotient (IQ) tests. According to Landy [34], the ability-based model of EI provides well-validated assessment instruments that limit the bias and other pitfalls commonly associated with self-report personality or trait-based measures of EI. The MEIT collects information about three branches of EI (perception, understanding and management) through 42 items and seven different tasks (i.e., perception and understanding branches are measured via two tasks each, while management is evaluated by a single task). The perception tasks assess the adequate perception of the own and others’ emotions through images and facial expressions; the understanding branch evaluates how emotions can coexist in particular situations, and how emotions can change in intensity over time; finally, the management tasks assess how participants react to achieve a desired outcome in an emotional situation. To obtain the total score, each branch contributes in the same way. The global reliability of the questionnaire using Cronbach’s alpha was 0.89, while the dimensions ranged between 0.85 and 0.91.

#### 2.2.2. Control Variables

Regarding sociodemographic data, we controlled age, gender, social status, educational level, job sector, type of working day, weekly working hours, years of work experience, and net monthly salary measured in euros, just as previous studies did [21,22,28]. Data about age, weekly hours of work, years of work experience, and earnings were collected through short-answer questions. The data about the type of working day (i.e., full-time or part-time), the labor sector (i.e., administration, education, etc.), educational level (i.e., school graduate, secondary school, university degree, etc.), and the social status (i.e., lower, middle, upper) was measured through multiple-choice questions. In order to perform the analyses, gender, educational level, and social class were coded.

### 2.3. Procedure

In line with previous studies, the professionals were recruited with the assistance of final-year psychology students who had previously been trained in the administration of questionnaires. The procedure was performed according to the guidelines provided by Wheeler et al. [35] to apply this sampling technique. The students contacted various associations of former university students, as well as companies from different sectors. The only exclusion criterion was part-time workers. The questionnaire was administered online using the Google Forms platform during 2020. The participants were informed of the voluntary and confidential nature of their collaboration. Once the questionnaires were answered, the information was stored in a database controlled by the research staff for subsequent statistical processing. This study adhered to the ethical guidelines mentioned in the Declaration of Helsinki and was approved by the Ethics Committee of the authors’ university (Universitat Jaume I, code: UJI-A2018-10). 

### 2.4. Data Analysis 

The data analyses were conducted using version 25.0 of the SPSS statistical package (SPSS Inc., Chicago, IL, USA). Cronbach’s alpha coefficient was used to determine internal consistency; bivariate correlations were obtained using Pearson’s r; lastly, a hierarchical linear regression analysis was performed to find out what percentage of the variance could be explained with each variable involved. The personal variables (i.e., age and gender) were included in the first step, whereas the socioeconomic ones were added in the second one. In the third step, the EI was added. These variables were added in that sequence in order to determine the extent to which EI and socioeconomic variables contributed to the prediction of salary beyond personal characteristics.

## 3. Results

### 3.1. Descriptive Statistics

Table 2 shows in detail which socioeconomic characteristics define the sample. Regarding the salary received, after grouping the results, we observed that the highest percentage is found in the range between EUR 1500 and 1800 (27.4%). The mean number of hours worked was 37.1 h (SD = 4.5), while the average years of work experience was 14.5 (SD = 3.7). 

### 3.2. Intercorrelations among Variables and Regression Analysis 

Table 3 shows the means, standard deviations, and correlations of the variables included in the study. Regarding the correlation coefficients between salary and EI, two significant correlations have been found. On the one hand, EI total (0.26) and on the other, EI management (0.27). In relation to the socioeconomic variables, a significant correlation is observed between salary and gender (0.26), between salary and level of studies (0.44), between salary and years of experience (0.16), as well as between level of studies and years of experience (−0.15). 

After establishing the significant relationship between variables, a hierarchical multiple-regression analysis was carried out to determine if EI could predict salary once the possible effect of individual and socioeconomic variables had been controlled. In the first step, gender and age were incorporated. In the next step, social class, educational levels, and years of experience were added in order to estimate the variance explained by the socioeconomic factors. Finally, the EI total and EI management were incorporated. Salary was introduced as the dependent variable. All predictors were mean centered prior to the analyses [36]. 

Table 4 shows the regression analysis in relation to the salary dimension. In the first step, the results indicate that both personal variables significantly contributed to the prediction of salary (R^2^ = 0.11; *p* < 0.01), as can be seen. Thus, being a man and an older adult was associated with higher salary than being a woman and younger. In the second step, the association between age and gender remained similar after including the socioeconomic variables. With regard to social class, education, and experience, the results showed a significantly positive association with salary (R^2^ = 0.23; *p* < 0.01). Thereby, belonging to a higher social class, reaching a high educational level, and having more years of work experience were associated with a higher salary. On the contrary, belonging to the lowest social class, having a lower level of education, and having few years of experience is associated with a lower salary. The second step increased the explained variance to 23% (∆R^2^ = 0.12; *p* < 0.01). Finally, EI was added in the third step. The previous associations continued to be the same. Regarding EI association with salary, the results showed a significant positive association (R^2^ = 0.33; *p* < 0.01) and a significant rise compared to the previous step (∆R^2^ = 0.10; *p* < 0.01). Therefore, having high EI and a good capacity for emotional regulation seems to be associated with receiving a higher salary, as had been hypothesized.

## 4. Discussion

Until now, scientific literature has pointed out the existence of important individual differences when using affective information, which has important consequences in the work environment [4,14]. In line with this, the present research analyzes the predictive capacity of EI on salary in a sample of multioccupational workers. Specifically, an attempt was made to determine whether EI and its dimensions contribute to the prediction of salary received by workers beyond the other variables already demonstrated.

The results of this study provide evidence about the importance of EI in predicting professional success, in this case evaluated as salary. Specifically, the participants who showed higher scores in the variables EI total and EI management also reported higher salaries. These data are in line with those found by prior research [14,22,23], which seems to indicate that people with higher emotional capacities obtain a higher salary. This phenomenon could be due to the role of EI in the construction of interpersonal relationships and the development of effective coping strategies, as proposed by Armstrong et al. [20]. EI helps us to use communication effectively and to develop cognitive processing of emotional information. Therefore, individuals with high EI should have stronger relationship-building skills, allowing them to become more deeply embedded in social networks. Such structural social capital provides access to resources and assistance from colleagues, leading to increased performance and higher compensation [21]. Regarding the emotional management dimension, other studies in which different measures of EI have been used, such as the MSCEIT [37], also found significant correlations between the ability to manage emotions and salary level [22,38]. Within the EI model of Salovey and Mayer, the management branch is mainly related to the effective management of one’s own and others’ emotions to achieve a desired result. In this sense, it is probable that those subjects with more ability to manage their emotional reactions modify their behavior more easily and quickly, which would help them better adapt to social situations within the work environment. This would help to explain the positive relationship found in this study. Regarding the branches of perception and understanding, although individually they do not show a significant relationship with salary, their influence when constructing the total IE variable indicates that they add value when they are taken into account together.

The data also indicate the predictive capacity of the variables gender, age, years of experience, educational level, and social class. The effect of gender continues the line of previous works [20,21] and the data provided by the Spanish National Institute of Statistics [32]. This could be attributed to several decisions related to the professional field as the greater tendency of women to leave work during the years of raising their children [39]. The influence of age and years of experience is also in line with the results of several previous investigations [21,23,28], which could be related to the usual development produced over the years [40]. Those employees with more experience achieve promotions and accomplish more important jobs, which are also better paid. As for the educational level, a positive relationship is also observed in reports by the Spanish National Institute of Statistics where salary increases as the educational level of citizens does [32]. It is expected that those subjects with a higher level of education will apply for higher positions, which leads to higher salary. The same phenomenon occurs when we observe the social class. There is a positive relationship between pertaining to the middle as well as upper social class and salary, however, this relationship is inverse for people of the lower social class. Social class, defined as one’s overall societal status and measured by indices of income, educational attainment, and occupational status, is remarkably stable across time and generations [41]. Therefore, it is logical to think that belonging to a higher social class facilitates having more job opportunities, partly due to the possibility to study for longer, at more prestigious institutions and having contacts in positions of power, which can positively affect the career development.

### 4.1. Limitations

The present work has several limitations that suggest future lines of research. First, the cross-sectional nature of the data makes it more difficult to establish the direction of the relationships between variables. Although the data is based on broad theory, replication of these findings through longitudinal studies would provide more information about the contribution of EI to salary. The second weakness is related to not having controlled the influence of factors such as IQ, as was done in previous studies [23]. Moreover, it would have been advisable to take into account the different personality dimensions since prior research shows that these have an influence on the results obtained in the work environment [24,26]. Specifically, it would have been relevant to take into account the dimensions of neuroticism and responsibility, because their influence on professional success has been observed [25]. Finally, there is a limitation regarding the sample, since the workers were selected for convenience through psychology students, which is a nonrandom technique with possibly limiting factors. Although this technique has demonstrated validity and reliability, as well as great utility in field studies within organizational psychology [35], this sampling method may be biased towards more cooperative participants, thus limiting the generalization of the results.

### 4.2. Practical Implications

This research, with its strengths and weaknesses, provides new evidence to understand the influence that EI has on work environments, specifically on salary, which is considered a key component in the development of individuals and companies [42]. Salary is not the only indicator of success, but it is a clear external sign of value within an organization and industry compared to other workers. Furthermore, salary is not subject to the perceptual biases of self-reported indicators of career success which can be influenced by various contextual factors and individual differences. Therefore, our findings have significant implications for curricula, employee selection, and development programs. In this regard, several authors affirm that EI could be improved through adequate training using scientifically based programs [43,44,45]. Therefore, it seems appropriate to implement this type of training to help people achieve greater socioemotional skills, which will contribute to professional success, achieving higher levels of well-being and better performance.

Concerning the novelty of this study and its contribution, while many claims have been made about the importance of EI for career success [14,15], our study establishes an empirical relationship between an ability-based measure of EI and an objective indicator of career success. In addition, our sample was composed by workers of all ages, unlike most research so far, which evaluated this relationship in recent graduates and younger workers. Finally, we also want to highlight that this study is the first to analyze the relationship between EI and salary, taking into account the social class and the educational level of the sample, which have proved to be fundamental variables to understand this connection.

## 5. Conclusions

In summary, this research underlines the importance of EI as predictor of individual salary in a multioccupational sample of Spanish workers. Those professionals with high EI have more resources to face the demands of their job, thus maximizing the outcomes. These results demonstrate the importance of EI in understanding individuals at work, and emphasize the necessity of developing socioemotional skills at early stages. Therefore, it is necessary to design and implement intervention programs in order to promote EI and develop healthy workers, which can prevent the development of mental diseases while helping them to reach their best possible performance.

## Figures and Tables

**Table 1:** Individual characteristics of the sample.

Characteristics	
Age (Mean, SD)	39.4, 10.9
Gender	(%)
Male	39.1
Female	60.9
Educational level	(%)
Basic	12.1
Intermediate	29.7
High	58.2
Social class	(%)
Lower	14.3
Middle	80.2
Upper	5.5
Occupational sector	(%)
Administration	24.6
Education	23.5
Healthcare	18.3
Commerce	12.1
Industry	11.8
Other sectors	10.1
Kind of employment	(%)
Private	47.6
Public	31.1
Self-employed	12.5
Unemployed	8.8

**Table 2:** Socioeconomic characteristics of the participants.

Characteristics	
Net salary (Mean, SD)	(1823, 199.1)
Net salary	(%)
• EUR 900 or less	6.6
• EUR 900–1199	17.9
• EUR 1200–1499	22.1
• EUR 1500–1799	27.4
• EUR 1800–2099	13.9
• EUR 2100 or more	12.1
Weekly working hours (Mean, SD)	(37.1, 4.5)
Years of work experience (Mean, SD)	(14.5, 3.7)

Note: *n* = 785.

**Table 3:** Mean, standard deviation and correlations between the study variables.

	1	2	3	4	5	6	7	8	9	10
1. Salary										
2. EI total	0.26 **									
3. EI Perception	0.11	0.76 **								
4. EI Understanding	0.05	0.79 **	0.59 *							
5. EI Management	0.27 **	0.82 **	0.66 *	0.60 *						
6. Gender	0.25 *	0.12	0.06	0.13	0.12					
7. Age	0.19 *	0.05	0.09	0.08	0.08	−0.02				
8. Educational level	0.42 *	0.18	0.11	0.02	0.03	0.11	−0.05			
9. Social class	0.45 **	0.12 *	0.10 *	0.14 *	0.17 *	0.03	0.07	0.26 **		
10. Years of experience	0.16 *	−0.07	−0.03	0.09	−0.08	0.05	0.28 *	−0.15 *	0.12	
Mean	1823	102.1	101.4	102	102.4	0.61	39.4	2.46	1.94	14.5
Standard deviation	199.1	14.6	15.3	14.6	15.7	0.41	10.9	0.76	0.19	3.7

Note: *n* = 785. Gender: 0 = male, 1 = female. Educational level: 1 = basic, 2 = intermediate, 3 = high. Social class: 1 = lower, 2 = middle, 3 = upper. * *p* < 0.05; ** *p* < 0.01.

**Table 4:** Multiple linear regression analysis for the salary variable.

Employees’ Salary			
Predictors ^a^	Step 1 ^b^	Step 2 ^b^	Step 3 ^b^
Age	0.17 *	0.16 *	0.11 *
Gender	−0.09 *	−0.07 *	−0.05 *
Lower social class		−0.30 **	−0.21 **
Middle social class		0.08 *	0.06 *
Upper social class		0.34 **	0.30 **
Basic educational level		−0.24 **	−0.20 **
Intermediate educational level		0.09 **	0.06 *
High educational level		0.20 **	0.17 **
Years of experience		0.16 **	0.11 *
EI total			0.23 **
EI management			0.17 *
R^2^	0.11 **	0.23 **	0.33 **
∆R^2^		0.12 **	0.10 **

Note: *n* = 785. ^a^ Gender: 0 = male, 1= female. Social classes were coded = 1, other social class = 0. Educational levels were coded = 1, other educational level = 0. ^b^ Standardized betas and probabilities: * *p* < 0.05; ** *p* < 0.01.
